# Visual Responses to Moving and Flashed Stimuli of Neurons in Domestic Pigeon (*Columba livia domestica*) Optic Tectum

**DOI:** 10.3390/ani12141798

**Published:** 2022-07-13

**Authors:** Shuman Huang, Xiaoke Niu, Jiangtao Wang, Zhizhong Wang, Huaxing Xu, Li Shi

**Affiliations:** 1 Henan Key Laboratory of Brain Science and Brain-Computer Interface Technology, School of Electrical Engineering, Zhengzhou University, Zhengzhou 450001, China; schuman@stu.zzu.edu.cn (S.H.); jointall@gs.zzu.edu.cn (J.W.); wzz1982@zzu.edu.cn (Z.W.); xuhuaxing@zzu.edu.cn (H.X.); shili@zzu.edu.cn (L.S.); 2 Department of Automation, Tsinghua University, Beijing 100084, China

**Keywords:** optic tectum, visual responses, encoding model, computational modeling of neural signaling

## Abstract

**Simple Summary:**

Avian can quickly and accurately detect surrounding objects (especially, moving ones). However, it was unknown how different neurons in the optic tectum (OT) in domestic pigeons (*Columba livia domestica*) processed moving objects compared to static ones. The electrophysiological results showed that the latency of response to moving stimuli was shorter than that to flashed ones, while the firing rates of response to moving stimulus were higher than that to flashed ones. Furthermore, the modeling study demonstrated that the faster and stronger response to a moving stimulus compared to a flashed stimulus may result from the accumulation process across space and time by tectal neurons. This study also sheds new light on the understanding of motion processing by birds.

**Abstract:**

Birds can rapidly and accurately detect moving objects for better survival in complex environments. This visual ability may be attributed to the response properties of neurons in the optic tectum. However, it is unknown how neurons in the optic tectum respond differently to moving objects compared to static ones. To address this question, neuronal activities were recorded from domestic pigeon (*Columba livia domestica*) optic tectum, responsible for orienting to moving objects, and the responses to moving and flashed stimuli were compared. An encoding model based on the Generalized Linear Model (GLM) framework was established to explain the difference in neuronal responses. The experimental results showed that the first spike latency to moving stimuli was smaller than that to flashed ones and firing rate was higher. The model further implied the faster and stronger response to a moving target result from spatiotemporal integration process, corresponding to the spatially sequential activation of tectal neurons and the accumulation of information in time. This study provides direct electrophysiological evidence about the different tectal neuron responses to moving objects and flashed ones. The findings of this investigation increase our understanding of the motion detection mechanism of tectal neurons.

## 1. Introduction

The visuomotor system of birds is highly accurate and precise and gives them the ability to judge the relative position of moving objects [1]. This ability is beneficial for them to avoid collisions with obstacles in flight or capture fast-moving prey [2] and may be attributed to the neuronal response properties in the optic tectum (OT). OT is responsible for the generation of orientation movements to the stimuli of interest, namely prey, predators and self-motion. Previous electrophysiological studies found that neural latency for moving bars was shorter than that for flashed ones [3,4,5]. However, these studies were mostly conducted in mammals. However, it remains to be investigated what was unique about OT neurons that encode moving objects. Further investigating response properties to moving compared to flashed objects may help us to deeply understand the underlying mechanisms of how birds capture fast-moving prey, which are very essential to their survival in complex environments [6].

Neuroelectrophysiological studies have reported some neuronal response properties to moving objects and flashed ones [3,4,5,7,8,9,10,11]. Originally, the phenomenon that neuronal response latency for moving bars was shorter than that for flashed ones was found in rabbit and salamander retinal ganglion cells [4]. Subsequently, investigations reported that the time-to-peak latency of population response to moving stimulus was shorter than that to flashed stimulus in the primary visual cortex of anesthetized cats [3] and awake monkeys [5]. Moreover, several studies reported that neurons in the mammalian primary visual cortex responded far more vigorously to moving stimuli than to stationary ones [12,13]. It is unclear whether this phenomenon also exists in the OT of birds. Previous statistical analysis has shown that approximately 60% of units in anesthetized pigeon OT responded more strongly to moving stimuli than to stationary ones [14]. Another study in vitro showed that wide-field tectal neurons were sensitive to dynamic stimuli but not to stationary stimuli, and further proposed a model of the tectal circuitry to predict its properties [15]. However, the conclusion of this study is based on the electrical stimulation of retinal ganglion cell axons and tectal cell dendritic endings, which is different from real visual stimulation and in vivo recordings. The motion detection mechanisms of one type of tectal neurons with motion-sensitive properties were investigated in detail based on a series of studies [15,16,17,18,19]. These neurons were usually sensitive to a continuous motion object but insensitive to stationary objects. In addition, there were other types tectal neurons with sensitivity to both moving objects and static ones. For such neurons, a hypothesis of energy cumulative computational strategy was raised in an encoding model [20]. This study only made a hypothesis and was an abstract published in a conference proceeding with limited results. Thus, the electrophysiological evidence is lacking about the different tectal neuron responses to moving objects and flashed ones, as is the detailed descriptions of the model to elucidate the potential computation performed by an individual neuron.

The electrophysiological experiments provide a simple, intuitive means for observing phenomena. The encoding models serve as effective tools to further reveal neuronal encoding mechanisms underlying certain neuronal phenomena, providing potential computational strategy. Generalized Linear Model (GLM), as a common encoding model framework, was always used to link the functional descriptions of visual processing with neural activity recorded across the visual system and described the encoding process in terms of a series of filters. Note that GLM was a statistic probabilistic model using multiple explanatory variables and these filters do not represent specific biophysical variables. However, this model has provided a basic framework that allows the addition of modules to represent new biological observations, and they can still provide insight into underlying biological processes in some cases. It has been used to characterize neural encoding in a variety of sensory, cognitive and motor brain areas [21,22,23].

Thus, we attempted to explore the differences in neural responses to moving objects and flashed objects by carrying out electrophysiological experiments and establishing an encoding model. We designed flashed square stimuli and moving square stimuli at different speeds, and synchronously recorded single unit’s activities from the middle and deep layers in an anesthetized pigeon OT with multielectrode arrays (MEA). We calculated and compared the first spike latency (FSL) and firing rate (Fr) at each location inside the receptive field (RF) from the responses to different types of visual stimuli. Furthermore, we adopted the encoding model based on the GLM framework by adding a spatiotemporal integration module, in which the integration of spatial and temporal information was realized in the time domain. Together, the insights gained in this study will help to further understand the mechanism of moving target detection by avian.

## 2. Materials and Methods

Nine adult domestic pigeons (*Columba livia domestica*) were used in this study. All pigeons (either sex, weighing 300–500 g) were supported by the animal center, and housed in accordance with the guidelines of the Care and Usage Committee. The study protocol was approved by the ethics review board (No SYXK 2019-0002).

**Figure 1:**
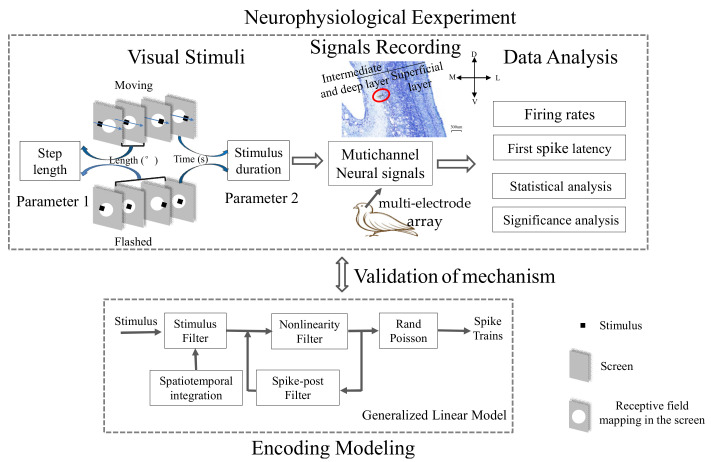
Schematic diagram depicting experimental design and data analysis. The pigeon was firstly restrained and the visual stimuli were presented. Neuronal signals were synchronously collected and the responses to moving and flashed squares were compared. Furthermore, we measured the dependence of response differences on different stimulus parameters. Finally, we adopted the encoding model based on the Generalized Linear Model framework by adding a spatiotemporal integration module, mimicking the spatiotemporal integration of tectal neurons. An example image of brain slices showed the implantation site of a recording unit.

The schematic diagram of the study showed details of the experimental step for comparison of the visual responses to moving and flashed stimuli of tectal neurons in pigeons (Figure 1), including animal preparation (see Section 2.1 for details), visual stimuli (see Section 2.2 for details), signal recording (see Section 2.2 for details), data analysis (see Section 2.3 for details) and modeling (see Section 2.4 for details).

### 2.1. Animal Preparation

Surgical techniques were similar to those previously described [24,25,26,27]. In short, the pigeon was initially anesthetized with urethane (1.8–2.2 g/kg) and restrained in a stereotaxic apparatus (model ST-5ND-B; Chengdu Instrument, Chengdu, China). A small dorso-lateral tectum region on the left side that could be easily accessed by a simple craniotomy was exposed, and the overlying dura mater was peeled [28]. The right eye was held open by removing the nictitating membrane and eyelids with surgical scissors and the left eye was covered. The neuronal data were all recorded with a multi-electrode array composed of 16 polyimide-insulated platinum/iridium microwires (Clunbury Scientific, Bloomfield Hills, MI, USA), which were arranged in four rows with four wires in each row: electrode spacing = 550 µm; row spacing = 250 µm; and impedance = 20–50 kΩ. The array was advanced approximately 1000–1200 µm below the tectum surface using a micromanipulator (Mc1000e; Siskiyou, San Diego, CA, USA). The experimenter intermittently monitored the anesthetized pigeons to assess their eye movements during data recording and no eye movement was observed [29]. During the experiment, the right eye was dripped with saline every half hour to keep the eye moist. Furthermore, the receptive fields of the recorded unit were obtained at intervals of 30 min by the experimenter, and no shift in RF location was observed.

**Figure 2:**
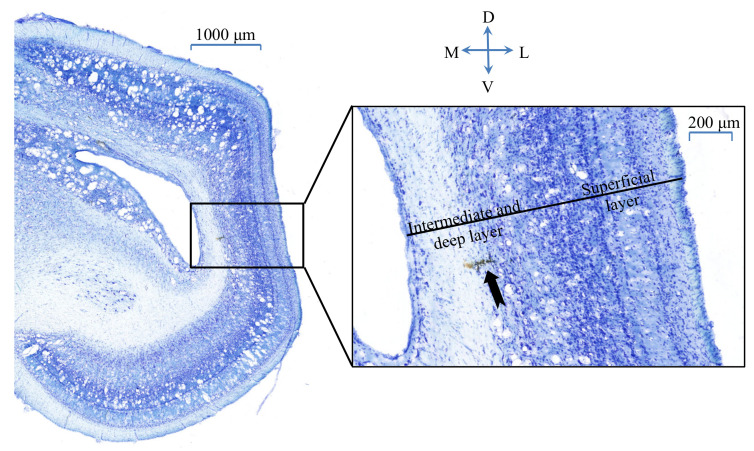
Representative photomicrograph of Nissl-stained coronal section showing the recording site of a sample unit. The right panel showed an enlarged view of the boxed area in the left panel. The black arrow pointed to the recording site. M—Medial; L—Lateral; D—Dorsal; V—Ventral.

The actual position of a recorded unit was located in the intermediate and deep layers of the OT. Verification of the target site was consistent with our previous study [27]. At the end of the experiments, the location of recording sites was marked by electrolytic lesions (200 µA for 10 s) of the brain tissue. Then, the pigeon was given an overdose of 20% urethane and perfused transcardially with saline followed by 4% paraformaldehyde. After the brain was removed, tissues were immersion-fixed for 12 h in 10% phosphate-buffered formalin, followed by immersion in a 30% sucrose solution for 24 h at 4 °C. The brain was cut to produce 40 µm-thick sections on a freezing microtome, which were identified in the Nissl-stained coronal sections (Figure 2).

### 2.2. Visual Stimulation and Electrophysiological Recordings

Visual stimuli were generated by a visual stimulus generator (ViSaGe MKII, Cambridge Research Systems, Rochester, UK) and displayed on a gamma-calibrated CRT monitor (Sony G520; Sony, Tokyo, Japan. Monitor size: 300 × 400 mm; resolution: 480 × 640 pixels; frame rate: 100 Hz), placed 40 cm in front of the pigeon’s right eye. All stimuli were presented on a window that is 300 mm × 300 mm (480 × 480 pixels) in the central region of the screen in the experiment. The size of the window region subtended 41.1° of visual angle viewed from 40 cm and each pixel is 0.08°.

**Figure 3:**
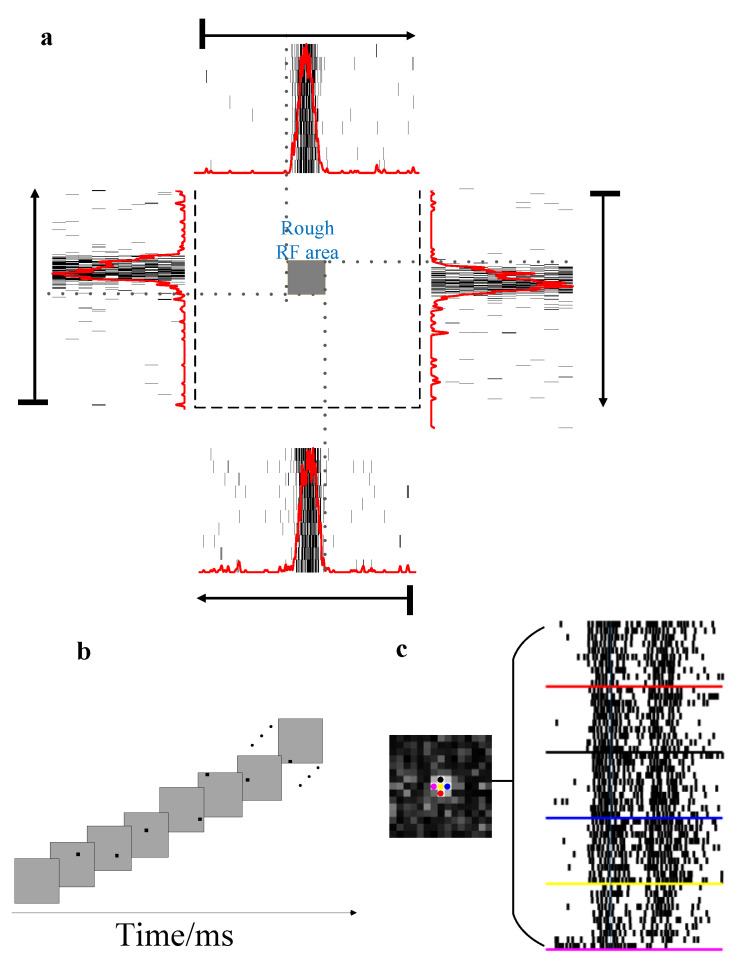
Receptive fields of a recorded unit. (**a**) Rough receptive field area was estimated with the paradigm of a long bar moving from one side to the other side over the screen. The adjacent surrounding arrows denote the moving direction of the bar. The outer surroundings present the firing spike train and post-stimulus time histograms of the moving bar in each direction for 20 repetitions. (**b**) Paradigm of sparse noise flashed inside the rough receptive fields area. (**c**) A detailed receptive field was measured, with the grey levels representing the number of spikes evoked by the stimulus, and then the bright area was selected as the center of the receptive fields. The right five subfigures show ten repeats of the firing spike train corresponding to the location indicated with the same color.

To rapidly and accurately map the receptive field, a long black bar of 0.5° in width and 40° in length moved with a speed of 8.33°/s on the screen in four directions (up, down, left and right) covering the whole screen to measure an initial rough estimation of the receptive field firstly (Figure 3a). Then, the above rough RF area was divided equally into 15 × 15 units in which sparse noise stimuli are presented for detailed mapping of the center of the RF, with a dark square flashing on bright background for 60 ms (time of a single flash at one position) in a pseudo-random sequence (10 flashes/position) at each position (Figure 3b,c).

**Figure 4:**
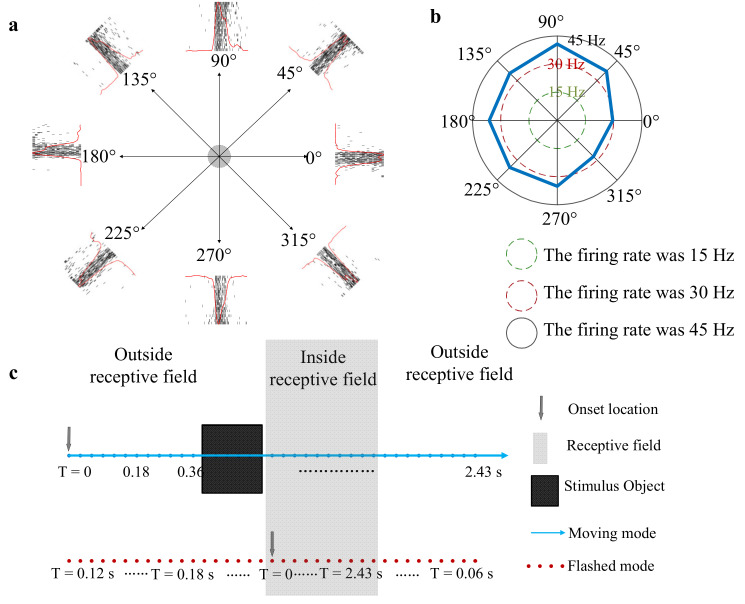
Directional tuning curves and paradigm of stimulus. (**a**) A square stimulus (stimulus A) moves in eight directions through the receptive field center. The shaded center (gray) is the receptive field center of the recorded unit. The arrows denote the moving direction of the square. The outer surroundings presented the firing spike train as well as its post-stimulus time histogram in 20 repeats of a moving square in each direction. (**b**) An example of tuning curves in eight directions. The blue line is the firing rate, and the green, red and black circles represent firing rates (spike counts in 1 s) of 15, 30 and 45, respectively. (**c**) An illustration of stimuli B and stimuli C in visual space. The grey area delimits the receptive field, with the square’s location marked by a black dot. A line of blue dots is the trajectory of motion stimulus and the arrow indicates the starting position of the moving square (stimuli B). A line of red dots is the location of the flashed stimulus, which is presented in a pseudo-random sequence (stimuli C).

After that, we designed the following three types of stimuli:

1. Stimulus A: A square (square: 0.1 cd/m^2^, background: 40 cd/m^2^) moved from one side to the other side across the RF area at 10°/s in eight directions (spaced by 45° with nasal 0°, Figure 4a) in a pseudo-random sequence (20 times/direction) to determine the preferred direction of a given unit. The length of the square side was equal to the radius of the measured RF and the inter-trial interval was 80 ms.
2. Stimulus B: A moving square (the same size as the square in stimulus (A) was presented on a gray background (40 cd/m^2^) (a line of blue dots in Figure 4c). The square luminance was 0.1 cd/m^2^. The length of the path (the length is three times as long as the length of the measured RF) remains consistent in the experiment. In the moving condition, a square was presented at random speeds in a specific direction (direction was determined by stimulus (A)). After one motion period finished, there was a 100 ms gray blank (40 cd/m^2^) followed to avoid adaptation [25]. The midpoint of the moving square’s trajectory was at the receptive field center. Furthermore, the moving speed was manipulated by the moving step and stimulus durations (time length of a single stimulus staying at one location). The moving step manipulation experiment and stimulus duration manipulation experiment were designed as follows. In the moving step manipulation experiment, the square was presented for two video frames (stimulus duration at one location: 20 ms) at each location, but the moving step length (the distance between adjacent blue dots in Figure 4c) was set to 0.08°, 0.24°, 0.4°, 0.8° and 0.96° (corresponding to speeds of 4°/s, 12°/s, 20°/s, 40°/s and 48°/s, respectively). Since the length of the total path remains constant, the duration of one motion period decreased as the speed increases. In the stimulus duration manipulation experiment, the moving step length was fixed at 0.24°, and the time length of stimulus presentation on each location (hereinafter referred to as the stimulus duration) was set to 10 ms, 20 ms, 30 ms, 40 ms, 50 ms and 60 ms, corresponding to speeds of 24°/s, 12°/s, 8°/s, 6°/s, 4.8°/s and 4°/s, respectively.
3. Stimulus C: A flashed square, which was the same size as the moving square, was presented in the same position in a pseudo-random sequence (20 times/position) that matched the moving square’s trajectory (a line of the red dot in Figure 4c). The time of a single flash at one position is constant with the time of a single square at one motion location. In the above motion paradigm, since motion is a continuous process, the square sequentially crossed different parts of the RF without any gray blank, which was always used to avoid adaptation, between adjacent locations. To keep stimulus conditions constant, there was also no gray blank between each two adjacent flashing squares in the flashed paradigm.

Neuronal signals were collected using a Cerebus 128-channel system (Blackrock Microsystems, Salt Lake City, UT, USA) and amplified 4000 times. The spikes were first extracted by band-pass filtering (second-order Butterworth) of the raw signals between 250 Hz and 5 kHz at a sampling rate of 30 kHz and detected with a threshold set at a signal-to-noise ratio of 1.50. Spike sorting was then performed to isolate single units by applying an unsupervised clustering algorithm [30]. The spiking events were saved for offline analyses, which were performed using MATLAB R2019a (The Mathwork).

### 2.3. Data Analysis

The border of each receptive field was estimated roughly using a bar moving across the visual display. The firing spike train was time binned (10 ms/bin) and the post-stimulus time histogram (PSTH) was obtained to observe a rough responding area (Figure 3a), in which the larger instantaneous firing rates were evoked than the baseline (quantified as the averaged firing rate in the same length of bin window before each stimulus onset). Second, the detailed RF area was measured by calculating the firing rate at each square on the screen grid for all units. The RF center was the position at which the highest firing rates were found. The RF size was determined in the same way introduced in the previous study [27]. The center site of the receptive field is zero. Invalid channels (a channel without a measured receptive field) are deleted.

After the RF was mapped, the temporal course of the response to each direction was observed by PSTH. Then, the mean spike firing rates in a given time window (equal to the duration of the square moving through the RF) under each direction were calculated to draw the directional tuning curves (Figure 4b). Thus, we chose the preferred direction (evoking the highest mean firing rates) for the following experiments.

To quantitatively compare responses to moving and flashed stimuli, the locations of given flashed squares were first aligned to the motion trajectory. Then, we extracted the temporal response feature (timestamp of the first spike fired, denoted by FSL) (Shadlen and Newsome, 1994) and response strength feature (the number of spikes in a given time window, represented with Fr) to the stimulus at each location.

Finally, we compared the response to the two paradigms under various speed conditions by taking the stimulus at the RF center as an example. The refractory period of the response lasted about 20 ms in our study, the time windows used to characterize the response to the 10 ms stimulus and 20 ms stimulus were both delayed by 20 ms. From the response of flash and moving bar under various conditions, we computed the following test statistics: FSL and Fr were derived from flashed and moving stimuli and the difference between flash and moving stimuli. The Wilcoxon signed-rank test was used for statistical analysis between the data of the same size and Wilcoxon rank-sum test was used for the data of different sizes. The *p*–values of the results were adjusted for multiple testing using the Bonferroni method with Bonferroni-adjusted significance threshold *p* < 0.05. The statistical comparison graphs were drawn using Origin 2019b.

### 2.4. Modeling

**Figure 5:**
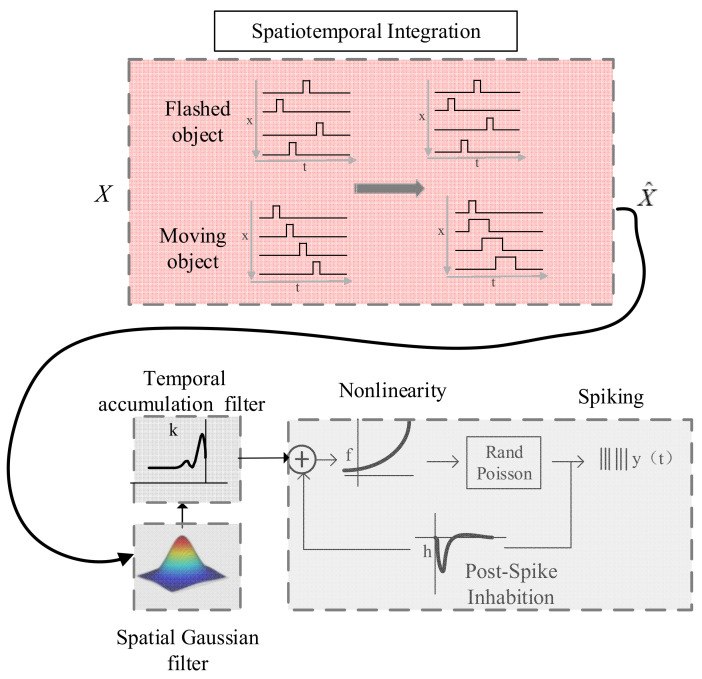
Schematic of the model structure. This model was based on Generalized Linear Model framework by adding a spatiotemporal integration module to stimulus filters. The spatiotemporally correlated stimulus input was firstly transformed into a temporally correlated stimulus. The outputs of three filters (a spatial filter, a temporal filter and a post-spike filter) were summed and passed through a nonlinear function *f* to determine the conditional intensity. Finally, spikes are generated via a conditionally Poisson process.

To further reveal the underlying information as to why neurons fired more spikes and responded faster to a moving stimulus rather than a flashed one, we hypothesized that the response difference between the moving and flashed stimuli may result from spatiotemporal integration mechanisms and adopted an encoding model based on the GLM framework by adding a spatiotemporal integration module (Figure 5).

Generalized Linear Model [22] was mainly composed of a stimulus filter *K*, which represented the integration of external stimuli, a post-spike filter $\vec{h}$, which captures the influence of spike history on the current probability of spiking, and a probabilistic spiking stage, which generated spike trains. Note that the stimulus filter of the original GLM always consisted of two independent filters, a spatial filter and a temporal filter. The spatial filter represented the spatial stimulus integration at a time and was usually fitted with Gaussian function (representing the spatial structure properties of the receptive field). The temporal filter represented the integration of historical stimulus on response to the current spatial position. Here, a spatiotemporal integration module was added to the stimulus filter to transform adjacent positions in space to adjacent time domains (red region of Figure 5). The transformation was defined as

(1) $$\hat{X}(x_{n},t_{m})=\left\{\begin{array}{cc} X(x_{n-1},t_{m}) & X(x_{n-1},t_{m})\neq 0\;\&\;X(x_{n},t_{m+1})\neq 0\\ X(x_{n},t_{m}) & \mathrm{otherwise} \end{array}\right.$$

where $X(x_{n},t_{m})$ and $\hat{X}(x_{n},t_{m})$ represented the stimulus and transformed stimulus in space $x_{n}$ at time $t_{m}$. X≠0 indicated the presence of visual stimuli. 

A neuron’s membrane potential was governed by the integration of stimulus and post-spike filter as follows:

(2) $$V_{t}=K({\vec{k}}_{x},{\vec{k}}_{t})\cdot \hat{X}(x,t)\cdot +\vec{h}\cdot {\vec{y}}_{hist}$$

where the stimulus filter K contained a spatial filter ${\vec{k}}_{x}$ and a temporal filter ${\vec{k}}_{t}$. $\vec{h}$ was the post-spike filter. ${\vec{y}}_{hist}$ was a vector representing spike history. The ${\vec{k}}_{x}$ was fitted with Gaussian function. ${\vec{k}}_{t}$ and $\vec{h}$ were fitted with a raised cosine basis in the form:

(3) $$b_{j}\left(t\right)=\left\{\begin{array}{cc} \frac{\cos\left(\log\left[t+d\right]-\phi_{j}\right)+1}{2a} & \frac{\log\left[t+d\right]-\phi_{j}}{a}\in \left[\begin{array}{cc} -\pi & \pi \end{array}\right]\\ 0 & \mathrm{otherwise} \end{array}\right.$$

where $\phi_{j}$ was evenly spaced from $\phi_{1}=\log\left(0.0+c\right)$ to $\phi_{10}=\log\left(0.1+c\right)$, so that the peaks of the filters spanned 0 ms to 100 ms.

Then, membrane potential passed through a nonlinear function $f_{r}$ to produce the spike rate $\lambda_{t}$ as the conditional intensity of a Poisson process.

(4) $$\lambda_{t}=f_{r}\left(V_{t}\right)$$

Finally, the spike trains were obtained by an inhomogeneous Poisson spiking process.


(5) $$y_{t}|\lambda_{t}\;\sim \;Poiss\left(\Delta \lambda_{t}\right)$$

Parameters of the established model were fitted with tectal neuron responses to a black square flashed or moving, recorded from an anesthetized pigeon with a microwire electrode array. The raster of predicted response was obtained from output of model. The firing rates were calculated by averaging the number of spikes observed in 1 ms bins, and then PSTH was calculated by smoothing with a Gaussian filter with a standard deviation of 30 ms.

The model performance was evaluated with residual error [31,32,33] (difference between the predicted and the recorded neuron data) and correlation coefficient between the predicted PSTH and the recorded neuron data. The residual error was calculated over nonoverlapping moving time windows [*m* − *n* ~ *m*] (*m* − *n* ≥ 1).

(6) $$e(t)=\left(\sum_{i=m-n}^{m}N_{i}-\int_{t_{m-n}}^{t_{m}}\lambda \left(t\right)\right)÷I$$

where $N_{i}$ denoted the firing rates of the recording unit in the *i*th time bin. $\lambda \left(t\right)$ indicated the predicted firing rates in each time bin, and *I* represented the total number of bins. The smaller residual error value represented the better performance of the model.

The correlation coefficient was defined as:

(7) $$\mathrm{rho}=\frac{\sum_{t=1}^{T}\left(PSTH_{Data}\left(t\right)-\bar{PSTH_{Data}}\right)\left(PSTH_{Model}\left(t\right)-\bar{PSTH_{Model}}\right)}{{\left\{\sum_{t=1}^{T}{\left(PSTH_{Data}\left(t\right)-\bar{PSTH_{Data}}\right)}^{2}\sum_{t=1}^{T}{\left(PSTH_{Model}\left(t\right)-\bar{PSTH_{Model}}\right)}^{2}\right\}}^{\frac{1}{2}}}$$

where $\bar{PSTH_{Model}}$ and $\bar{PSTH_{Data}}$ denoted the average value of the PSTH. The value of the rho ranges from −1 to +1, where ±1 indicated a total positive or negative correlation and 0 indicated no correlation.

To examine whether differences in neural responses were captured by the model, the FSL and Fr obtained from the model output with flashed input were compared to those with moving input. Furthermore, the fitted temporal filter and spatial filter were shown to analyze the biological plausibility of the model.

## 3. Results

A total of 40 recorded units from the OT of nine pigeons were used in the study. The average RF size of all recorded units was 8° ± 1° in diameter. Note that approximately 87.5% (35/40) neurons significantly responded to stimulus A, regardless of the moving direction and sequence of the stimuli. Thus, these neurons had no motion direction selectivity. For this reason, we specified one direction evoking the highest mean firing rates in Figure 4b in the following experiments. The following statistical results were all based on the 35 units. For each part of the following results, we only presented the detailed results of an example from a single recording site.

### 3.1. Response of OT Neurons (Unit) to Moving and Flashed Stimuli

**Figure 6:**
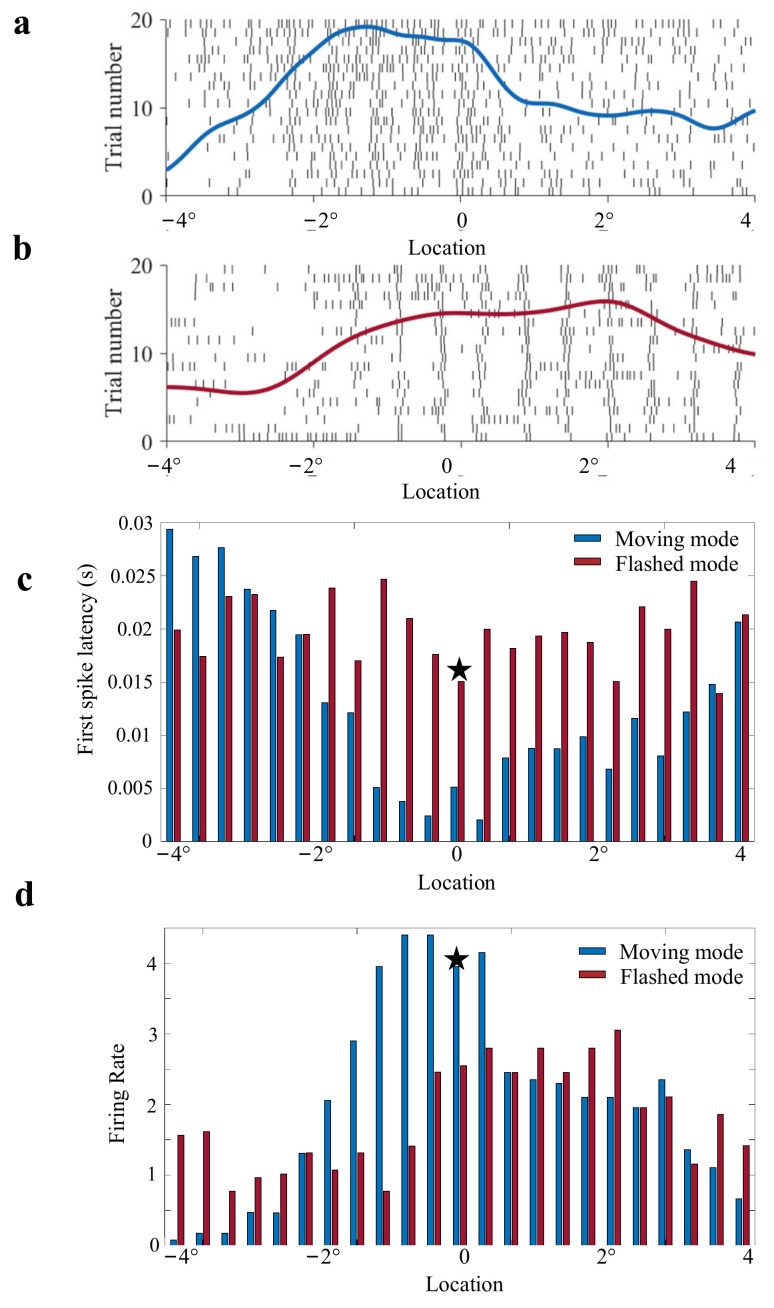
Responses to flashed and moving squares. (**a**) Raster plot showing neural responses to the moving stimuli, aligned to the stimulus onset time. The dot denotes a spike event and each row is a trial (only a subset of trials is shown). The blue line denotes the mean post-stimulus time histograms for 20 repetitions. (**b**) A raster plot and post-stimulus time histograms for the flashed stimulus. The responses were sorted by location sequence. (**c**) The histogram of the first spike latency to moving and flashed square stimuli at a location inside the receptive field for 20 repetitions. The blue histogram represents the response to a moving stimulus and the red histogram represents the response to a flashed stimulus. (**d**) The histogram of firing rates to moving and flashed square stimuli at a location inside the receptive field for 20 repetitions. The blue histogram represents the response to a moving stimulus and the red histogram represents the response to a flashed stimulus. Asterisk indicated a response to stimuli which crossed the receptive field center.

The responses of OT neurons obtained from moving and flashed stimuli were firstly compared. The RF size of an example unit was 8°. Since the center site of the RF was zero, the boundary sites of RF were −4° and 4°. The spike trains and their normalized PSTHs under 20 repeats presented a multiphasic response with an initial burst followed by a plateau and a decay in both moving and flashed conditions (Figure 6a,b).

Then, the calculated FSL and Fr at each location inside RF in moving stimuli and flashed stimuli were showed (Figure 6c,d). The blue bar represented the response feature obtained from the motion paradigm and the red bar represented those from the flashed paradigm. Neural responses are usually most stable at the center site of RF. The characteristics of response to stimuli at the RF center (the center site of RF was marked by an black asterisk in Figure 6c,d) were selected to be compared by referring to previous studies [3,4,5]. The comparison results showed that the latency of response to the moving stimulus was shorter than that to the flashed stimulus, whereas the firing rates in the motion paradigm were higher than those in flashed paradigm. These results suggested that the response to the moving stimulus was faster and stronger than that to the flashed stimulus.

### 3.2. Dependence of Response Differences on the Moving Step Lengths

**Figure 7:**
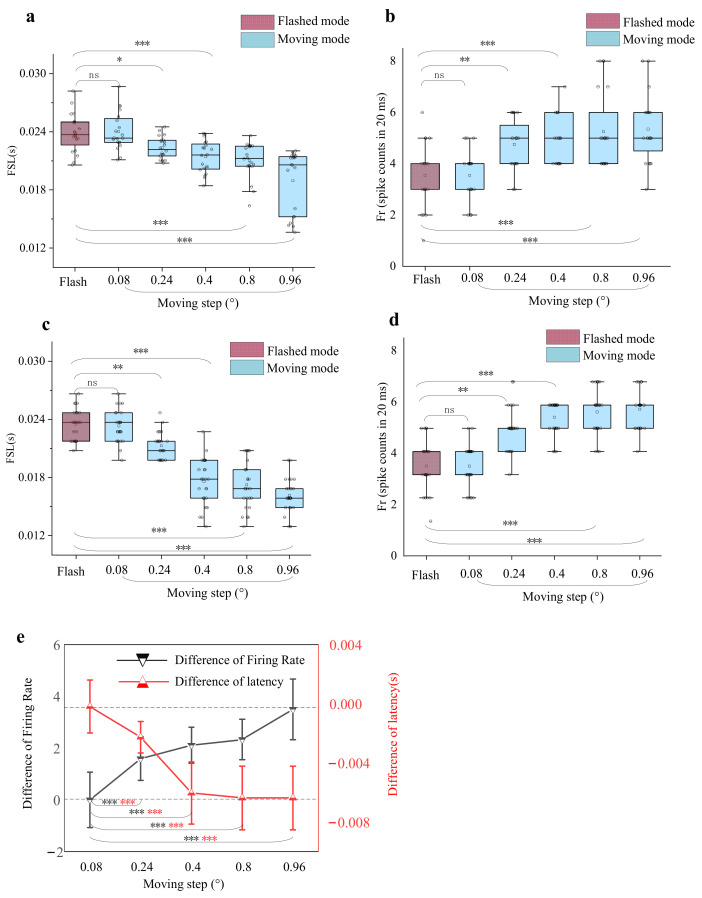
The results for varied moving steps. (**a**,**b**) The statistical results of the first spike latency and firing rates derived from different moving step length stimuli and flashed square stimuli for 20 repeats. The horizontal line indicates the median of each group of data and the whiskers indicate the lowest and highest point within 1.5× the interquartile ranges of the lower or upper quartile, respectively. The ‘ns’ indicates no significant difference between two groups of data (Bonferroni corrected, Wilcoxon rank-sum test, *p* > 0.05), ‘*’ indicates a weak difference between two groups of data (Bonferroni corrected, Wilcoxon rank-sum test, 0.01 < *p* < 0.05), ‘**’ indicates a little difference between two groups of data (Bonferroni corrected, Wilcoxon rank-sum test, 0.001 < *p* < 0.01) and ‘***’ indicates a significant difference between two groups of data (Bonferroni corrected, Wilcoxon rank-sum test, *p* < 0.001) (**c**,**d**). The boxplot graphs for statistical first spike latency and firing rates results of 27 recording units. Error bars as in (**a**,**b**). (**e**) The mean difference between Fr and FSL (flash latency minus motion latency) varied moving steps. All data represent mean (triangular symbols) and standard error (error bars), and dotted reference lines represent zero. Red asterisks indicate the levels of significance obtained from the mean difference in FSL. Black asterisks indicate the levels of significance obtained from mean difference in Fr.

The dependence of response difference on the flashed and moving stimuli was tested with moving step length. Twenty-seven units were used for moving step manipulation experiments. The calculated FSL and Fr based on response within five moving step lengths (0.08°, 0.24°, 0.4°, 0.8° and 0.96°) in the moving paradigm were compared with those in the flashed paradigm (Figure 7). The red box represented the response obtained from the flashed paradigm, and the latter five blue boxes (the length of moving steps from left to right were 0.08°, 0.24°, 0.4°, 0.8° and 0.96°, respectively) indicated those from the moving paradigm. The statistical results of 20 trials from the example unit showed that when the moving step was much shorter (0.08°), corresponding slow speed, FSL and Fr under flashed stimuli were not significantly different from those of moving stimuli (*p* > 0.05, Bonferroni corrected, *n* = 5, Wilcoxon signed-rank test). However, as the moving step grew a little larger, the latency of responses to the moving stimulus was shorter than that to the flashed stimulus (0.001 < *p* < 0.01 for moving step length of 0.24°, and *p* < 0.001 for moving step length of 0.4°, 0.8° and 0.96°, Bonferroni corrected, *n* = 5, Wilcoxon rank-sum test), while the Fr to moving stimulus was higher than that to flashed stimulus (0.01 < *p* < 0.05 for moving step length of 0.24°, and *p* < 0.001 for moving step length of 0.4°, 0.8° and 0.96°, Bonferroni corrected, *n* = 5, Wilcoxon rank-sum test). The statistical test analysis for the recorded 27 neurons (average response of each neuron over 20 trials) under different stimuli paradigms (Figure 7c,d) indicated that all recorded units exhibited consistency (*p* > 0.05 for moving step length of 0.08°, 0.001 < *p* < 0.01 for moving step length of 0.24° and *p* < 0.001 for moving step length of 0.4°, 0.8° and 0.96°, Bonferroni corrected, *n* = 5, Wilcoxon rank-sum test). As the moving step length increased, the latency differences between the moving stimuli and flashed ones grew, and so did the firing rate differences (Figure 7e, *p* < 0.001 Bonferroni corrected, *n* = 4, Wilcoxon rank-sum test).

### 3.3. Dependence of the Response Difference on Stimulus Duration

**Figure 8:**
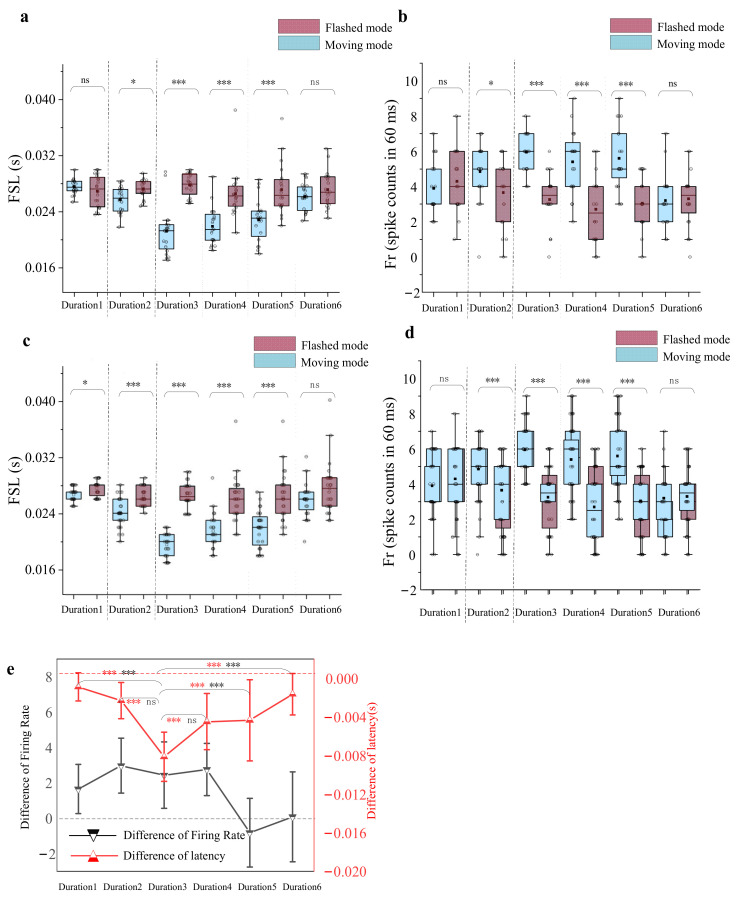
Results of varied stimulus durations. (**a**,**b**) The example results of the first spike latency and firing rates derived from two paradigms under varied duration paradigms for 20 repetitions (stimulus duration was set to 20 ms and moving direction was set to right). From left to right, the stimulus durations were 10 ms, 20 ms, 30 ms, 40 ms, 50 ms and 60 ms. The ‘ns’ indicates no significant difference between two groups of data (Bonferroni corrected, Wilcoxon rank-sum test, *p* > 0.05). ‘*’ indicates a weak difference between two groups of data (Bonferroni corrected, Wilcoxon rank-sum test, 0.01 < *p* < 0.05) and ‘***’ indicates a significant difference between two groups of data (Bonferroni corrected, Wilcoxon rank-sum test, *p* < 0.001). (**c**,**d**) A statistical result of 28 recording units. The boxplot graphs for first spike latency and firing rates derived from varied duration paradigms. (**e**) Mean difference in first spike latency and firing rates (flash latency minus motion latency) varied stimulus duration. All data represented mean (triangular symbols) and standard error (error bars), and dotted reference lines represent zero. Red asterisks indicated the levels of significance obtained from the mean difference in FSL. Black asterisks indicated the levels of significance obtained from the mean difference in Fr.

The dependence of the response difference on the flashed and moving stimuli was further tested with stimulus duration. A total of 28 recording sites were tested in the stimulus duration manipulation experiment. The FSL and Fr with different stimulus durations (10 ms, 20 ms, 30 ms, 40 ms, 50 ms and 60 ms) were calculated and further compared between flashed and moving stimuli (Figure 8). The comparison results of 20 trials for an example recording unit (Figure 8a,b) showed that (1) the response latency to moving stimulus was shorter than that to flashed stimulus for the durations of 20 ms (0.01 < *p* < 0.05, Wilcoxon rank-sum test), 30 ms, 40 ms and 50 ms (*p* < 0.001, Wilcoxon rank-sum test), while there was no significant difference between the two stimuli for the durations of 10 ms and 60 ms (*p* > 0.05, Wilcoxon signed-rank test). (2) The Fr derived from the moving stimulus was higher than that of flashed stimulus in the durations of 20 ms (0.01 < *p* < 0.05, Wilcoxon rank-sum test), 30 ms, 40 ms and 50 ms (*p* < 0.001, Wilcoxon rank-sum test), rather than 10 ms (*p* > 0.05, Wilcoxon signed-rank test) and 60 ms (*p* > 0.05, Wilcoxon rank-sum test). Furthermore, the statistical results of 28 recording units (Figure 8c,d,e) showed that the difference derived from flashed and moving stimuli increases first and then decreases as the stimulus duration grew larger (Difference in latency: *p* < 0.001; Difference in firing rate: *p* > 0.05 for durations of 20 ms and 40 ms, *p* < 0.001 for durations of 10 ms, 50 ms and 60 ms, Wilcoxon rank-sum test).

Taken together, the above results showed that the response to a moving stimulus was faster and stronger than that to a flashed stimulus, and the response differences were not only dependent on the moving step length between adjacent locations, but also dependent on the stimulus duration at each location.

### 3.4. Modeling of Spatiotemporal Integration Mechanisms

**Figure 9:**
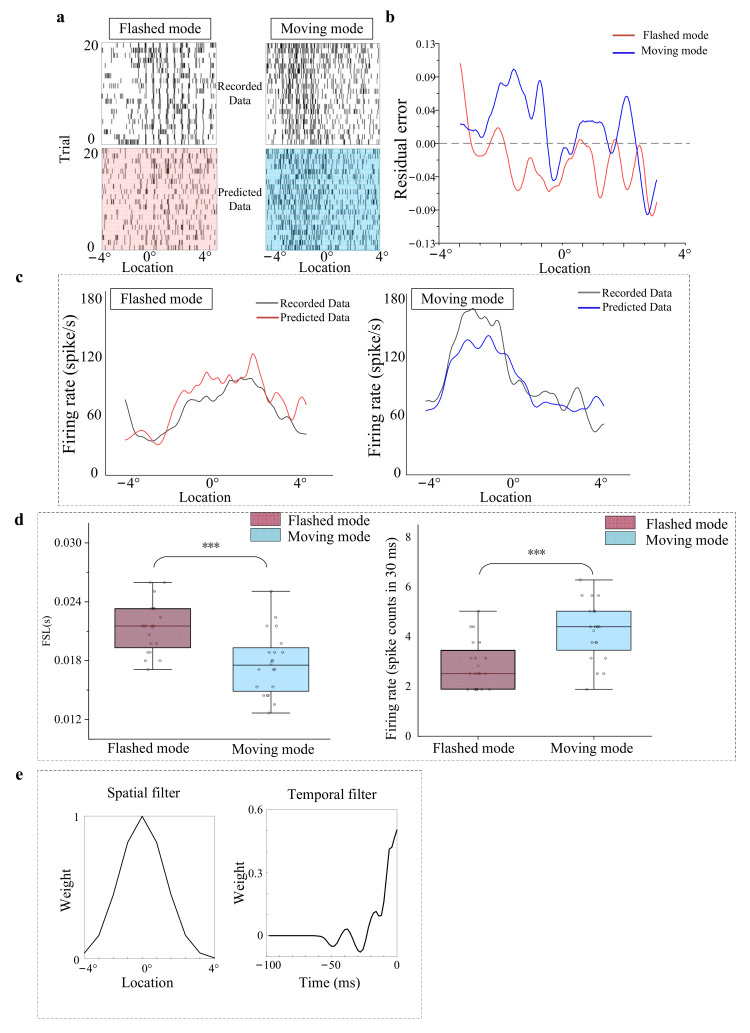
Simulation results of the model. (**a**) Raster plot of real neural responses and predicted neural data to the flashed stimuli (left) and moving stimuli (right). (**b**) The residual error between the recorded data and the predicted response to the flashed stimuli (red) and moving stimuli (blue). (**c**) The corresponding post-stimulus time histograms for 20 repetitions for the flashed paradigm (left) and the moving paradigm (right). The red line indicates true post-stimulus time histograms, and the gray line indicates predicted post-stimulus time histograms. (**d**) First spike latency (left) and firing rates (right) for moving and flashed stimuli generated by the model. The ‘***’ indicates a significant difference between two groups of data (Wilcoxon rank-sum test, *p* < 0.001). (**e**) Estimated spatial filter (left) and temporal filter (right) for the example recordings.

The comparison results between the recorded and predicted neuronal responses illustrated that this model could capture the structure of the spike trains evoked by flashed and moving stimuli to a certain degree (Figure 9a). The relatively small residual error ([−0.12~0.12], Figure 9b) suggested that the model is relatively good at predicting neural responses. The correlations between PSTHs (Figure 9c) of recorded and predicted data were calculated and indicated that the neural data predicted by the established model presented a significant correlation with the recorded responses to moving (0.81 ± 0.05, *p* < 0.001) and flashed stimuli (0.79 ± 0.08, *p* < 0.001). Furthermore, FSL and Fr of the predicted responses to moving and flashed stimuli were calculated. The statistical results of 20 trials (Figure 9d) were compared and showed that the response to a moving stimulus predicted by the encoding model was also faster and stronger than the predicted response to a flashed stimulus (*p* < 0.001, Wilcoxon rank-sum test). The results were more consistent with the recorded response, suggesting that our model could capture the difference between the response to moving and to flashed stimuli. Finally, the spatial filter and temporal filter (Figure 9e) suggested that the model was biological plausible. The shape of the spatial filter was consistent with the spatial properties of the receptive field. The temporal filter was positive at the time bin ([0 ms, 20 ms]), mimicking the integration of adjacent historical stimuli. The accumulative integration of spatial and temporal information was realized in the time domain.

## 4. Discussion

The results of the present study indicated that tectal neurons responded more accurately and quickly to moving stimuli than to flashed stimuli. The difference between responses to moving and flashed stimuli depended on moving step length and stimulus duration, both of which are related to the speed of the stimuli. This suggested that tectal neurons could integrate stimuli across the space and time domain. Furthermore, the encoding model based on the Generalized Linear Model framework combined with spatiotemporal integration module reproduced the shorter FSL and larger Fr of response to moving stimuli compared to flashed ones, implying an accumulation process across the space and time domain performed by tectal neurons in encoding targets that move along continuous trajectories, corresponding to the sequential activation in space and the accumulation of information in time domain.

Some studies have reported that neural latency for moving bars was shorter than that for flashed ones in the primary visual cortex and retinal neurons in mammals [3,4,12,13] and birds [14]. Here, we found that this phenomenon also exists in the pigeon optic tectum. In this study, our results show that the response to a moving stimulus was not only faster but also stronger s compared to a flashed stimulus. The stronger response to a moving stimulus may be explained by the cumulative effect, which was validated by an encoding model. Meanwhile, previous work has shown that stronger response was always accompanied by shorter latencies [34]. A combination of shorter spike latency and higher firing spike frequency assured the quick behavioral responses to moving stimuli. What is more intriguing, the higher firing rates of primary visual perception neurons could transmit salient information to the higher cortex for further processing, which can help them easily find food or locate the stealthy approach of a predator [35]. Although the similarities in mammals and birds indicate their neurobiological basis seem to share with mammals, evolutionary divergence is shaped by various environmental constraints and survival requirements, suggesting that these distinct encoding strategies are based on separate brain mechanisms. Note that, these neurons in our study were sensitive to motion and static objects, unlike these previous studies [15,16,17,18,19], which focus on the motion-sensitive neurons with sensitivity to motion but insensitivity to stationary objects. Our work thus complements existing studies about the motion detection mechanism of tectal neurons.

Furthermore, we analyzed the dependence of response difference to the flashed and moving stimuli on moving step length and stimulus duration. On one hand, our data showed that the difference between flash and motion conditions increased with moving step lengths. However, in contrast to our results, the latency difference between flash and motion conditions decreased with speed in mammals [5,36]. This difference may be due to the existence of various conduction velocity groups in the optic tract of the pigeon [37] and network of the avian midbrain [38,39,40]. The midbrain network has been demonstrated to play a key role in saliency computing and multimodal integration [38,41,42]. However, this conclusion needs further evaluation. In addition, it is unexpected that no significant difference was observed at small moving steps (Figure 7a), because small moving steps meant small luminance changes in adjacent sites or frames. Neurons in the OT were reported to be sensitive to changes in brightness within the RF [26]. Thus, small moving steps would induce a relatively weak response in the neurons to both moving and flashed stimuli.

On the other hand, the results of our study showed that the response difference of tectal neurons increased first and then decreased as the stimulus duration grew larger. The role of stimulus duration has been extensively studied in the visual, auditory, olfactory and tactile sensory systems [43,44,45,46]; however, the detailed effects of stimulus duration have not been understood. In our study, the stimulus duration influenced the continuity of motion. Long stimulus duration at each location (such as 60 ms in our study) may lead to the apparent perception of motion as if the movement was not smooth, but visually flashed. Thus, neurons were equally evoked by both moving and flashed squares. Moreover, a short stimulus duration may be not enough to induce the response of neuron in 10 ms, which leads to unreliable quantitative response (FSL and Fr).

Finally, our model could capture the detailed difference in neuronal response to moving and flashed stimuli and confirmed our hypothesis that the sequential activation of new dendritic endings in space conclusion by moving stimulus would cause the accumulation of activation in time conclusion. Previous work only implied that the cellular properties may depend on the particular sequential activation of the neuron’s dendritic endings [15]. However, this model lacked an explanation of the detailed difference between the neuronal response to moving and flashed stimuli. Our model supplemented the present understanding of existing encoding models. Furthermore, Bagheri presented a biologically inspired target tracking model which partially exploits facilitation, a slow build-up of response to targets moving along long, continuous trajectories in the dragonfly brain [47]. This led us to think that our study might be able to provide biological evidence for constructing a new moving target detection model or enhancing the performance of the existing model.

Several limitations warrant attention and should be addressed in future research. Our conclusion was drawn from urethane-anesthetized birds. Urethane, a common anesthetic for acute electrophysiological recordings in birds [26,48,49] has minimal effects on synaptic transmission while maintaining a surgical level of anesthesia [50,51]. Therefore, anesthesia might not introduce much bias to our analysis. Nevertheless, it would be more meaningful to investigate this issue towards awake and freely behaving birds. In addition, the sex differences in data collection were not taken into account in these experiments. Although the sex of animals was usually not considered in the related study [6,52,53], it may be more convincing to compare these results for each gender. Finally, the used model was biologically plausible to a certain degree, but it simplified the neural structure. Thus, neurostructural evidence is needed to determine that the spatiotemporal integration relates to such neuronal response properties.

## 5. Conclusions

We compared the response properties of tectal neurons in pigeons obtained from motion and flashed paradigms. The results showed that the response to a moving stimulus was faster and stronger than that to a flashed stimulus, and the response differences depend on the moving step length between adjacent locations and the stimulus duration at each location. Furthermore, a hypothesis of the spatiotemporal integration process by tectal neurons was proposed and the neuronal responses were imitated by an encoding model based on the GLM framework. Our findings provide new insights into the fast detection mechanism for moving objects by avian. In further studies, it would be interesting to explore the structural basis for these findings and measure the roles of the neural response properties in awake animals.

## Data Availability

The datasets analyzed in the current study are available from the corresponding author upon reasonable request.
